# Materials, Preparation Strategies, and Wearable Sensor Applications of Conductive Fibers: A Review

**DOI:** 10.3390/s22083028

**Published:** 2022-04-15

**Authors:** Xiuhong Li, Shuang Chen, Yujie Peng, Zhong Zheng, Jing Li, Fei Zhong

**Affiliations:** School of Mechanical Engineering, Hubei University of Technology, Wuhan 430068, China; 20200005@hbut.edu.cn (X.L.); 102010106@hbut.edu.cn (S.C.); pengyj1024@163.com (Y.P.); zhengzh215@163.com (Z.Z.); lijing@hbut.edu.cn (J.L.)

**Keywords:** conductive fibers, preparation strategies, wearable sensors

## Abstract

The recent advances in wearable sensors and intelligent human–machine interfaces have sparked a great many interests in conductive fibers owing to their high conductivity, light weight, good flexibility, and durability. As one of the most impressive materials for wearable sensors, conductive fibers can be made from a variety of raw sources via diverse preparation strategies. Herein, to offer a comprehensive understanding of conductive fibers, we present an overview of the recent progress in the materials, the preparation strategies, and the wearable sensor applications related. Firstly, the three types of conductive fibers, including metal-based, carbon-based, and polymer-based, are summarized in terms of their principal material composition. Then, various preparation strategies of conductive fibers are established. Next, the primary wearable sensors made of conductive fibers are illustrated in detail. Finally, a robust outlook on conductive fibers and their wearable sensor applications are addressed.

## 1. Introduction

Stretchable wearable devices have attracted extraordinary attention with the upsurge of interest in health monitoring systems and noninvasive human–machine interfaces. As a significant element of wearable electronics, a flexible sensor exhibits an unprecedented potential in human–machine interaction, human healthcare monitoring, electronic skin, artificial intelligence technology, etc. [1], which is capable of detecting and quantifying diverse bioinformation (body temperature, blood pressure, and respiratory patterns) with high specificity and sensitivity [2]. To accommodate the life expectancy of humans, lots of wearable sensors with diverse functions appeared [3]. Likewise, a variety of materials, such as metal sheets, metal wires, foam sheets, and plastic films, are employed to prepare wearable sensors [4,5,6,7,8]. Among them, the most popular and promising materials are conductive fibers owing to their advantages of superior flexibility, conductivity, breathability, durability, washability, biocompatibility, and so on, [3,9,10,11]. With recent advances in materials science and micro/nanofabrication, there has been active research on conductive fibers for wearable sensors.

Conductive fibers have broadly gone through three generations: (1) the first generation is flexible conductive fibers with traditional metals as raw materials, and the metals are designed to be stretchable structures, but with poor wearability, conductive instability, and other problems; (2) the second generation uses polymers as elastic conductive materials. Although their stability and conductivity are not ideal, the preparation process is simple and designable. Moreover, the material sources are wide. (3) The third generation is based on special textile yarns to fabricate composite elastic conductive fibers [1,12,13,14]. Currently, many conductive fibers manufactured by combining textile technology, mechanical technology, materials science, and electronics exhibit several advantages. Firstly, such conductive fibers have good flexibility and large deformation when being subjected to a very small external force, and their Young’s modulus ranges from MPa to KPa. Plus, they usually possess a large specific surface area of about 10^2–3^ m^2^/kg as well as varying degrees of porosity up to 99%, leading to good permeability [15]. In addition, some conductive fibers possess good durability, which can remain stable under stretching, bending, twisting, and shearing at various frequencies for more than 10,000 cycles. Furthermore, other conductive fibers can work not only at room temperature, but also under extreme temperature conditions. For example, some conductive fibers can work normally at low temperatures of −268.15 °C and others can operate at high temperatures of 250 °C [16,17,18]. It is known that the normal working temperature range of the majority of conductive fibers far exceeds the operating temperature range requirements of wearable sensors. Finally, these conductive fibers show good biocompatibility and do no harm to sensitive skin. Therefore, they have been made as artificial organs instead of necrotic organs [19,20,21,22,23]. Based on the building blocks of conductive fibers, they can be classified into three categories: metal-based conductive fibers, carbon-based conductive fibers, and polymer-based conductive fibers. Each conductive fiber has its own advantages: metal-based conductive fibers have relatively high conductivity, carbon-based conductive fibers have relatively low cost, and high fraction-based conductive fibers can be prepared to make more functional sensors.

**Figure 1:**
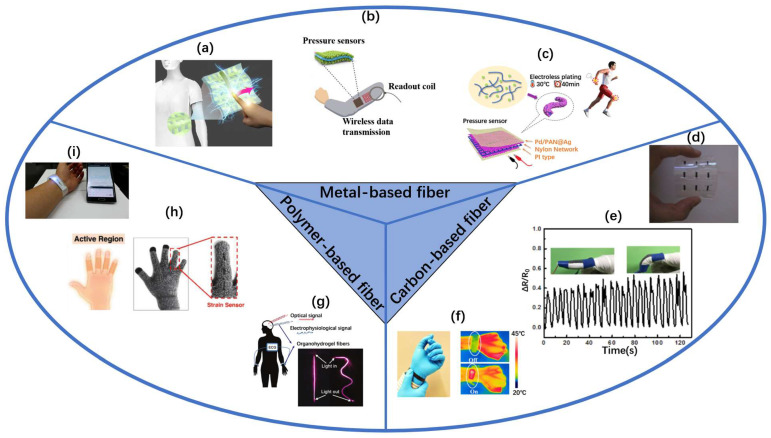
Various wearable sensors prepared from three types of conductive fibers used to detect various parameters of the human body: (**a**) capacitive sensors used in clothing [21], (**b**) an all-fabric pressure sensor with a wireless battery-free monitoring system [22], (**c**) piezoresistive sensor for monitoring human movement [23], (**d**) 3 × 3 flexible strain sensing array [24], (**e**) strain sensor monitors human movement [25], (**f**) a sensor that can be heated and monitored [26], (**g**) smart wireless blood pressure sensor [10], (**h**) a strain sensor applied to gloves [27], (**i**) capacitive sensor made into keyboard [28].

Due to the excellent features of conductive fibers mentioned above, it is perfect for them to be applied in wearable sensors, which are able to extract various signals and analytes, such as pressure, tension, humidity, temperature, etc., making it possible to monitor human health conditions [12]. Besides, wearable sensors made of conductive fibers are very soft and flexible so that they can be bent, pulled, and folded like textiles. In addition to the soft mechanical properties, the favorable biocompatibility enables them to fit well with human skin, making them particularly comfortable to wear. In terms of the superior advantages of conductive fibers, the wearable sensors made of conductive fibers can be generally divided into three main categories: pressure sensors, strain sensors, and other types of sensors according to the different applications. Pressure sensors, including resistive, capacitive, and piezoelectric, are to convert external mechanical variations to electrical signals, which is mainly used for monitoring human health condition. As one of the next-generation electronics, strain sensors composed of conductive fibers have been widely investigated owing to their excellent mechanical flexibility and stretchability compared to the traditional rigid strain sensors. Nowadays, they can be used in a variety of areas, such as electronic skin, smart textiles, soft robotics, and so on. Meanwhile, other types of sensors consisting of conductive fibers are gas sensors and humidity sensors. They are usually applied to detect toxic gases or control the temperature. All in all, no matter which type of sensors, they take advantage of the conductive fiber’s extraordinary features. Until now, lots of studies have been published to demonstrate the diverse raw materials and wearable sensors used and made for conductive fibers, respectively (as shown in Figure 1).

Obviously, conductive fibers show superior chemical and physical properties, so they can be promising materials for fabricating wearable sensors. To facilitate researchers to prepare many more kinds of conductive fibers with various functions for wearable sensors, a systematic summarization related to conductive fibers is presented. The work mainly focuses on three aspects of conductive fibers: materials, preparation strategies, and wearable sensor applications. First, the materials of conductive fibers used for wearable sensors are reviewed, which can be classified into three types: metal-based, carbon-based, and polymer-based conductive fiber. Then, the preparation strategies of conductive fibers are concluded. One is using conductive materials, such as polymers, to form a solution or melt to co-mingled spinning; the other is applying physical and chemical manner on (or within) the fiber surface to deposit and coat conductive substances. Next, the performances of the two typical wearable sensors containing pressure sensors and strain sensors are analyzed. At last, the future prospect of conductive fibers based on wearable sensors is discussed. This review will give a path for researchers to prepare an appropriate conductive fiber for wearable sensors with various functions for different applications.

## 2. Conductive Fiber

Owing to recent advances in materials science and micro/nanofabrication, conductive fibers play an increasingly essential role in the field of flexible wearable electronics because of their excellent properties mentioned above. They are usually composites of an external protective layer, a conductive layer, a connecting layer, and an elastomer. Among them, the protective layer, such as polydimethylsiloxane (PDMS), poly(styrene-b-butadiene-b-styrene) (SBS), etc., is composed of flexible cleaning-resistant material [29,30,31,32]. It is to keep the conductive layer from damage, which may decrease the conductivity of the conductive layer. Besides, the protective layer can help to increase the cleaning resistance of the conductive layer. Obviously, the conductive layer consisting of highly conductive material is responsible for electric conduction, mainly to achieve the conversion between mechanical and electrical signals, such as liquid metal, silver, carbon black (CB), polyacrylonitrile (PAN), and so on. Meanwhile, the role of the connection layer made of the adhesive material (polymethacrylate adhesive) is to firmly connect the conductive layer and the elastomer together, avoiding the conductive layer off in the process of deformation. However, there is no need for a connecting layer in conductive fiber where the conductive layer and elastomer can be directly and firmly connected with each other. The elastomer is primarily to provide strong mechanical strength for the conductive fibers, which are made up of superior flexible material, such as polyurethane (PU), polyethylene terephthalate (PET), PDMS, etc. Herein, we classify conductive fibers into three types: metal-based, carbon-based, and polymer-based conductive fibers based on the used raw materials which play a major role in conducting electricity [33,34,35]. The performance parameters of some conductive fibers are shown in Table 1.

### 2.1. Metal-Based Conductive Fiber

Metals, as the most common conductive materials in life, show high mechanical strength, thermal conductivity, and electrical conductivity of about 5 × 10^5^ S cm^−1^ [49]. For example, EGaIn, silver, aluminum, copper, nickel, and others are very suitable for making conductive layers of metal-based conductive fibers, and they have been successfully prepared as various conductive fibers with superior electrical conductivity [50,51,52]. Among them, liquid metal (EGaIn) has become a popular material to prepare metal-based conductive fibers due to its low melting point (29.8 °C), low viscosity, high surface tension, high electrical conductivity (3.4 × 10^4^ S cm^−1^), and good thermal conductivity [25,53,54]. It can be well combined with various elastomers, such as PDMS and PU, which can be manufactured as a flexible resistive strain sensor, followed by capacitive pressure sensors and piezoelectric pressure sensors [55,56,57,58]. Though the cost of EGaIn is very high, it is still highly favored by researchers. In a study, Liu et al. [59] injected EGaIn into hollow PDMS fibers to prepare a liquid-metal-based conductive fiber with high conductivity and excellent tensile properties. This conductive fiber was used to develop a resistive strain sensor. Silver is also often prepared as a metal-based conductive fiber by depositing silver nanowires or nanoparticles onto elastic fibers (e.g., nylon, PU fibers), which is mainly in the form of silver nanowires or silver nanoparticles [60]. Yan et al. [61] integrated AgNWs on top of PU fibers to form a conductive fiber with high conductivity and good stability. The conductive fiber was successfully prepared as a resistive strain sensor with high sensitivity. During the preparation process, the most important thing is to ensure that the coating is uniform, continuous, and thin, maintaining good electrical conductivity. The silver-based conductive fibers are generally prepared as a resistive strain sensor. Plus, there are also metals such as aluminum, copper, and nickel that have been applied as raw materials for the metal-based conductive fiber [62,63,64]. Those metals are mainly employed as plating layers combined with various elastomers, whose cost is relatively low compared to EGaIn and silver. However, copper and nickel would reduce the conductivity of the obtained metal-based conductive fibers due to oxidation, affecting the performance of the corresponding wearable sensors. In general, metal-based conductive fibers exhibit good electrical conductivity, durability, thermal conductivity, and high mechanical strength, which are suitable for manufacturing into functional wearable sensors, such as resistive strain sensors, resistive pressure sensors, piezoelectric pressure sensors, capacitive pressure sensors, etc. [65,66,67,68].

### 2.2. Carbon-Based Conductive Fiber

The development of carbon-based materials has attracted much attention in recent years due to their potential applications in a wide range of fields, such as energy storage devices, fuel cells, sensors, and electromagnetic shielding. Carbon and its derivatives are used in the manufacture of conductive fibers because of their remarkable characteristics, such as low cost, high electrical conductivity, large specific surface area, excellent chemical stability, and good mechanical durability [69]. The most commonly used carbon and its derivatives are graphene (G), graphene oxide (GO), reduced graphene oxide (rGO), CNTs, activated carbon (AC), and CB [70]. Among them, CB is in the form of black powder, with particle diameters ranging from 10 to 100 nm [71]. Compared with other types of carbon materials, CB is often employed for preparing carbon-based conductive fibers due to its wide sources. In the preparation process of carbon-based conductive fibers, CB is mainly dispersed uniformly in a solution and then coated on the surface of elastomers or it is mixed with the elastomer to form a solution or melt which is then cured into carbon-based conductive fibers. For example, Souri et al. [72] prepared a conductive ink using CB as the main raw material and applied it to elastic cotton fabric to produce conductive fibers. Such conductive fibers are usually made as resistive strain sensors and resistive pressure sensors [73]. CNTs are allotropes of carbon, which are also often used to fabricate carbon-based conductive fibers. However, CNTs have certain toxicity, which is super harmful to humans. Thus, safety precautions need to be taken when using CNTs to manufacture carbon-based conductive fibers [74]. Usually, CNTs are connected with the surface of the fiber to obtain conductive fibers [11]. Sometimes, CNTs as conductive fillers are put into hollow elastic fibers to prepare carbon-based conductive fibers [75]. In a study, Zhou and his colleagues prepared a highly stretchable conductive fiber by uniformly filling CNTs inside thermoplastic elastomer (TPE) tubes [62]. Similarly, G, GO, rGO, AC, etc., are used in a comparable way to prepare carbon-based conductive fibers [76,77,78,79]. For example, Souri et al. [80] fabricated a type of carbon-based conductive fiber by mixing G nanosheets into conductive inks and then coating the mixer onto cotton fabrics. In terms of safety, G nanomaterials are safer compared with CNTs [75]. In addition, it possesses better mechanical flexibility than metal nanomaterials [10]. In general, carbon-based conductive fibers possess superior advantages of electrical conductivity, chemical and mechanical durability, and low cost, which can be applied to make resistive strain sensors, resistive pressure sensors, humidity sensors, etc. They play a key role in the conductive fibers, resulting in significant parts in the field of functional wearable sensors.

### 2.3. Polymer-Based Conductive Fiber

Currently, there are many types of polymers, some of which have become the material choices for manufacturing multifunctional fibers or films owing to their high electrical conductivity, good processability, lightweight, high elasticity, and strong corrosion resistance [65,66]. Typical polymers used for conductive fibers are PPy, PANI, polythiophene (PTh), PEDOT: PSS, etc. [81]. Their conductivity is usually between 10^−8^ and 10^2^ S cm^−1^ at room temperature [82]. Commonly, the polymer-based conductive fibers are prepared by solidifying a co-blended solution of polymers and elastomers. Sometimes, the monomers of the conductive polymer are polymerized directly on the surface of the elastomer to form the conductive fiber. A few polymer-based conductive fibers are made via directly coating the conductive polymer on the elastomer surface, as with some metal-based conductive fibers. For example, Tadesse and his colleagues prepared the conductive paste PEDOT: PSS as the main materials. The conductive paste was then added to the appropriate amount of rheology modifier to increase the viscosity of the conductive paste. Finally, the conductive paste is coated on the fiber surface to form a polymer-based conductive fiber [29]. Polymer-based conductive fibers are often fabricated as resistive strain sensors, resistive pressure sensors, capacitive pressure sensors, piezoelectric sensors, etc. In addition, polymers including PPy, PAIN, PTh, etc., show different degrees of sensitivity to various gases (NH_3_, N_2_, CO, etc.), resulting in being developed as gas sensors. For instance, a polymer-based conductive fiber can be made into a wearable gas detection sensor, which uses PAIN and PAN nanofibers as the main raw materials. It possesses excellent sensitivity, fast response and recovery time, good reproducibility, and stability for NH_3_ at 10–2000 ppm at room temperature [83]. In summary, polymer-based conductive fibers are composed of a variety of polymers, which are not only prepared as resistive strain sensors, resistive pressure sensors, and capacitive pressure sensors, but also as diverse gas sensors [84,85]. This is an advantage that metal-based conductive fibers and carbon-based conductive fibers do not have.

## 3. Preparation Strategies

Conductive fiber, an important component of flexible sensors, can be prepared in several methods, which are briefly categorized into two main ways. One is the use of conductive materials to form a solution or melt to co-mingled spinning, such as electrospinning and wet spinning. The other is the use of physical and chemical manner to deposit and coat conductive substances on (or within) the fiber surface, such as dip-coating, chemical plating, electroplating, and so on. Additionally, sometimes a combination of the above methods is employed to fabricate conductive fibers, such as a combination of electrospinning and dip-coating.

### 3.1. Blended Yarn

Blended yarn is to blend two or more fibers together to form a yarn. This method is commonly used in the textile industry, and the researchers apply this method to the preparation of conductive fibers, mainly by mixing the metal fibers (or wires) and elastic fibers into a bundle of composite fibers, in which metal fibers or wires are used to achieve electrical conductivity. Qu et al. [30,86,87] used stainless steel (SS) fibers and polyester fibers as the main materials to be blended into yarns by a draw frame machine. Usually, the blended yarn method is conducted through three steps: firstly, different fibers are put into different jars at the entrance of the draw frame machines, and the machines will mix these fibers into a bundle in a certain ratio and store them in the exit jar, and, finally, the blended yarns can be obtained after a series of processing by other machines. They found that the blended yarns show the same SS fiber weight ratio, and the conductivity of the hybrid yarn with higher thread density is lower than that with lower thread density. The reason for this phenomenon may be that some of the SS fibers inside the yarn are more likely to break and form a fracture in the process of preparing the yarn with a higher thread density. Overall, the blended conductive yarn has good electrical conductivity, abrasion resistance, and moisture absorption, and is ideal for making flexible, comfortable, and wearable fabric sensors.

### 3.2. Dip-Coating

Dip-coating is a simple and efficient preparation process of conductive fibers, which usually involves three steps: firstly, the fibers are immersed in the conductive coating; secondly, the fibers are removed after a period of time; and, finally, the fibers are dried [88]. Zhang et al. [89] used a special liquid metal with a very thin and viscous oxide film on the surface to fabricate conductive fibers. During the dip-coating procedure, the elastic fibers are immersed inside the liquid metal bath, and then, after taking the fibers from the liquid metal bath, highly elastic liquid-metal-based composite fibers with an extremely thin liquid metal oxide film (about 5 μm thick) are obtained. As a special metal, liquid metal is often used as a conductive coating due to its liquid property. Chen et al. [16] also applied liquid metal (EGaIn) as a coating to prepare conductive fibers. The specialty of this study is that they applied a coating polymethacrylate (PMA) between PU fibers and liquid metal, which can adhere well to the surface of PU fibers and form hydrogen bonds with the oxide film of liquid metal, so PMA can well combine the oxide film on the surface of liquid metal and PU fibers [90]. Thanks to this intermediate layer, the liquid metal is uniformly distributed on the surface of PU fibers, and the obtained conductive fibers possess good durability under cyclic stretching. Chen et al. [47] employed AgNWs suspension as a coating to obtain AgNWs conductive fibers by immersing the PU fibers in AgNWs suspension, removing PU fibers and drying PU fibers, and cycling this process several times. Many researchers have used this approach to make conductive fibers with different properties for numerous functions. For example, they have prepared various conductive fibers, such as intrinsic conductive polymer PEDOT with polyelectrolyte poly (styrene sulfonate) (PSS), silver nanowires, graphene, CB, and so on [91,92,93,94]. These different functional conductive fibers can eventually be made into various types of wearable sensors.

### 3.3. Spray-Coating 

**Figure 2:**
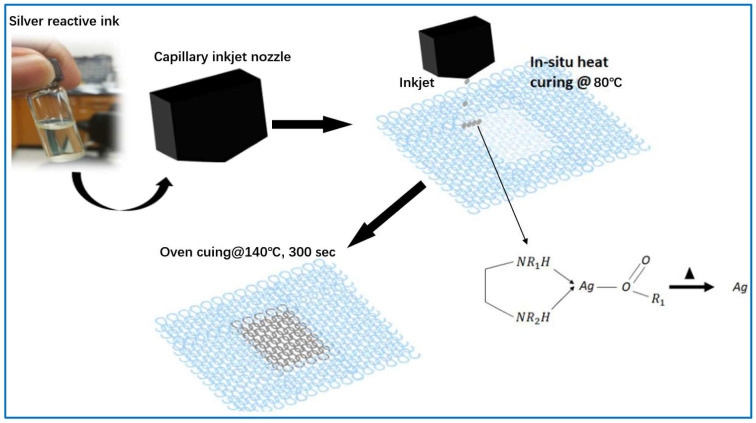
Schematic diagram of spray-coating [95].

Spray-coating is to use conductive materials to make conductive coatings, which are then, via the spraying system, uniformly covered to the surface of a single fiber or fiber woven to form conductive fibers or conductive textiles, respectively. The standard process of spray-coating is shown in Figure 2. Zahid et al. [95] used PEDOT: PSS conductive polymers and graphene to prepare a conductive coating, which was sprayed onto the fibers to obtain a conductive, breathable, and lightweight mercerized cotton fabric. Jo et al. [19] have successfully obtained a silver nano conductive fiber by using silver nanowires sprayed on polyester fibers through a supersonic spraying device, which promotes the adhesion and efficient deposition of the nanowire network by physically fusing the joints.

In addition, researchers have discovered high conductive inks, which are great raw materials for printing. To solve the problem of clogging of inkjet printer nozzles by nanoparticle conductive ink, a new, reliable, and conformable particle-free active silver ink is created. It uses amine compounds to form particle-free solutions with dissolved complex ions of silver salts, which will react to produce silver when heated to a certain temperature. This ink is printed onto the fiber and then heat cured to obtain a silver-coated conductive fiber. However, this ink has some limitations of material choices. Because the reactive silver ink only shows good adhesion to the functional groups of polyester and polyamide surfaces, this results in restricted applications [96]. In order to make the conductive ink adhere well to carbon fibers, Fogel et al. [27] fabricated conductive inks with epoxy resin masterbatches and carbon nanotubes, which were uniformly sprayed onto a carbon fiber grid laid by carbon fibers, and, finally, cured into stretchable conductive carbon fibers. Moreover, researchers have successfully manufactured conductive inks with different functions and good adsorption properties through experimental studies, thus breaking the limitations of conductive inks mentioned before, allowing them to be integrated into textiles, ceramics, glass, polyethylene, polypropylene, paper, metal, wood, and so on [97]. The preparation of conductive fibers using spray-coating displays advantages of simplicity, flexibility, and efficiency. 

### 3.4. Chemical Plating 

**Figure 3:**
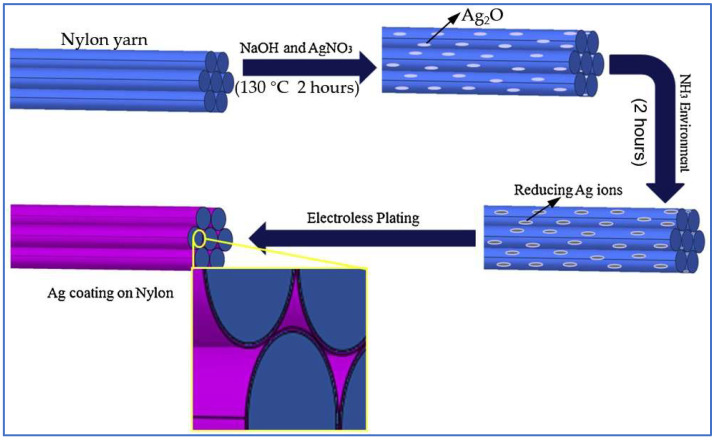
Schematic of preparation process of the flexible nylon/Ag conductive fiber via chemical plating [35].

Chemical plating is a way of preparing conductive fibers using the chemical principle of redox in the absence of electricity, where the metal ions are reduced to metal deposited on the surface of the fiber to form a dense coating by adding a reducing agent to a solution containing metal ions [47]. The common plating layers are silver, aluminum, nickel, and copper, which provide high electrical conductivity to the fibers. Owing to the very good electrical conductivity of silver, some researchers prepare silver-plated conductive carbon fibers by plating a layer of silver on the surface of carbon fibers. Due to the impurities and smooth texture as well as the hydrophobic and inert surface of carbon fibers, the process of silver plating will hinder the chemical reaction of hydrophilic compounds so that the carbon fibers need to be purified, sensitized, activated, rinsed, and undergo other pretreatments before the silver plating. Lu and colleagues have made a silver-plated conductive fiber with good electrical conductivity and thermal stability via the following steps: firstly, the treated carbon fiber was immersed in the HCHO solution. Then, an appropriate amount of AgNO_3_/ammonia solution was added and stirred for one hour at room temperature, and the silver ions were successfully reduced to silver precipitates, which were uniformly deposited on the carbon fiber surface. Finally, the silver-coated carbon fibers with good electrical conductivity as well as thermal stability were obtained after cleaning [98]. Other groups have used a similar method to plate elastic fibers with silver from a salt solution to obtain various silver-plated composite fibers [99]. Similarly, Qureshi et al. prepared a flexible nylon/Ag conductive fiber via chemical plating, as shown in Figure 3 [35]. Although silver possesses an excellent electrical conductivity, its price is high, so researchers choose to apply the relatively inexpensive aluminum to replace it as the plating layer. Choi and colleagues obtained aluminum-plated conductive fibers by choosing PET fibers and (AlH_3_{O(C_4_H_9_)_2_}) ink as the target fibers and aluminum precursor, respectively [100]. Furthermore, Gao et al. and Lai et al. have also employed an electroless plating manner to prepare nickel-coated carbon fibers [13] and copper-coated PBO fibers [101], respectively. All of these conductive fibers mentioned above have good electrical conductivity and high elasticity, which are excellent materials for making wearable sensors.

### 3.5. Electroplating

Electroplating, also called electrodeposition, is a common way to prepare a conductive fiber through the electrolytic effect, where the plated metal cations in the salt solution get electrons to generate metal monomers deposited on the surface of the target fiber to form coating. The thickness of the plated layer can be controlled by changing the current level and the deposition time, and the common plated metals are nickel, copper, silver, and so on. Researchers prepared a conductive fiber by electroplating a layer of nickel on the surface of the carbon fiber. For example, Shin et al. [36] used this method to coat cotton textile surface with nickel. As a common metal in life, copper is often used as a coating. Ali et al. coated the copper on the surface of nylon fibers with concentrated sulfuric acid (H_2_SO_4_) and the copper plate as the cathode and the anode, respectively. They found that the copper-coated fibers show a good durability verified by a corrosion resistance test [102]. Thanks to the high-conductivity property, silver is naturally made into silver-coated fibers by researchers. Liu et al. [103] applied a two-step method to prepare the silver-coated polyester fiber with better mechanical properties and washability by chemical plating and electroplating. In order to obtain multi-functional conductive fibers, a layer of material will be coated on the outside of the former coating. Tran et al. used sputtering to coat gold on the surface of carbon fibers, and then used electroplating to coat copper on top of the gold coating. Since the sputtered gold layer acts as an inner layer, the wettability and reactivity of the carbon fiber surface are enhanced, thereby improving the uniformity of copper electrodeposition. With the prolongation of the deposition time, the thickness of the copper layer increases, and the electrical properties of the carbon fiber/gold/copper composite conductive fiber are remarkable [42]. Electroless plating is an excellent way to fabricate conductive fibers. First, it can change the thickness of the coating by controlling the current level and the length of time. Second, the composite conductive fibers with multi-layer coatings can be obtained by replacing the coating raw materials and performing multiple electroplating. 

### 3.6. In Situ Polymerization

**Figure 4:**
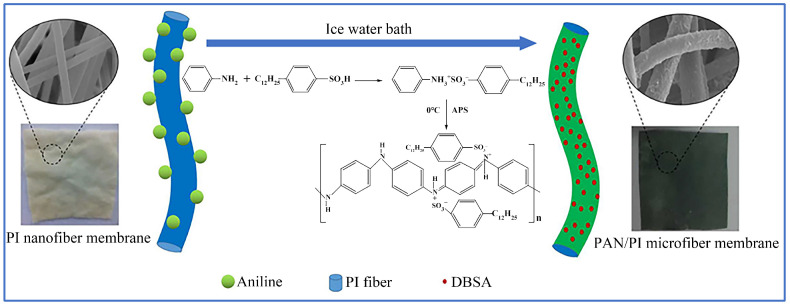
Schematic of preparation process of the PANI/PI microfiber membranes via in situ polymerization [107].

In situ polymerization for the preparation of conductive fibers is a process of forming polymer chains on the surface of the fiber, which shows two advantages. One is that the method has little effect on the mechanical properties of the fiber. The fibers can retain the air permeability and flexibility after polymerization because the process occurs around each fiber. The other one is that the mechanical bond between fibers and polymers is strong so that the polymer will not separate from the fiber during deformation [104]. Xie et al. [105] reported a PPy cotton fabric exhibiting high electrical and thermal properties and strong mechanical strength by in situ polymerization. The preparation process was conducted by the following steps. Firstly, the untreated cotton fabric was immersed in the aqueous solution of pyrrole. Then, an ice bath was carried out, while the oxidant chloride hexahydrate (FeCl_3_-6H_2_O) was added into the solution. As a result, the polymerization occurred on the cotton fabric and the PPy was generated. Finally, the cotton fabric was removed and dried in the oven to obtain PPy cotton fabric. PANI is one of the most promising conductive polymers with advantages such as simple preparation, good environmental stability, and high electrical conductivity. Tissera et al. [106] prepared PANI cotton fabric by polymerization with an aniline monomer on the cotton fabric. As shown in Figure 4, Wang et al. [107] successfully utilized the same monomer and dodecylbenzene sulfonic acid (DBSA) to obtain PANI/PI conductive microfiber membranes by the same method. The DBSA is used as a functionalized organic proton dopant to enhance the performance of PANI.

### 3.7. Chemical Vapor Deposition (CVD) 

**Figure 5:**
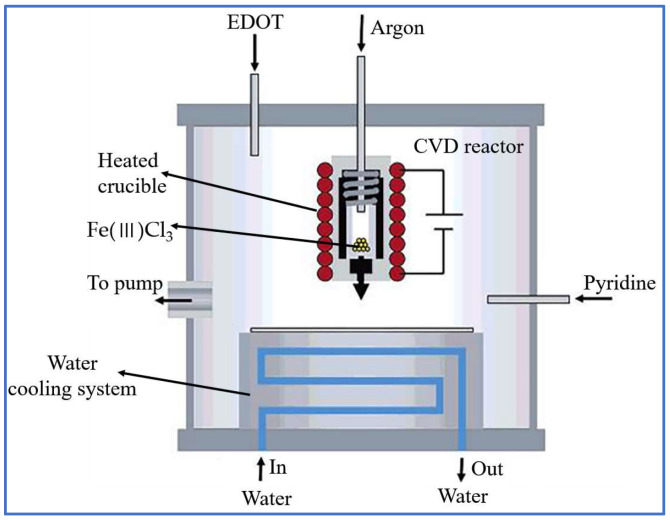
Schematic of preparation process of the conducting PEDOT films via CVD [111].

CVD is a process that utilizes the chemical reaction of gases near a substrate to deposit various materials. In a typical CVD process, the substrate is exposed to one or multiple volatile precursors, which react or disintegrate on the surface of substrate to form the deposited materials [108]. This approach is widely used in the semiconductor industry, as it can provide a solid coating with high quality and resistance on any substrates [109]. Among the conductive fibers used in portable and wearable electronic products, graphene fibers are one of the most popular because of their small size, good flexibility, and excellent wearability [110]. For instance, Wang et al. [40] prepared conductive graphene fibers by CVD. They twisted a plurality of copper wires into a bundle used as a catalyst and put the bundle into a quartz glass tube. A mixture of hydrogen and argon gas is applied to remove the oxidized material from the copper surface, and then ethanol vapor is passed to the reactor for a period time. When the reactor is cooled to room temperature, the graphene can be seen. The PMMA (4 wt% anisole in solution) is coated on the surface of the graphene grown on the copper wire, which is as a support to strengthen the graphene structure. After removing the copper wire and PMMA, the conductive fibers can be obtained. Sun et al. [73] used a similar method to prepare conductive graphene fibers with copper as a catalyst. Except for graphene fibers, PEDOT elastic fibers with good flexibility and wearability are also often fabricated by CVD. Gleason et al. and Bashir et al. have directly deposited PEDOT on the surface of viscose fibers by polymerizing EDOT monomers in the gas phase, as shown in Figure 5 [111]. CVD is a versatile economical process for preparing conducive fibers because it can be applied to deposit any elements or compounds and coat them on several different parts at the same time. However, CVD sometimes employs toxic, corrosive, flammable, and explosive precursor gases, resulting in certain safety issues [112].

### 3.8. Physical Vapor Deposition (PVD)

PVD is a promising technology for functionalization of fibers, which is a basic vapor coating process meaning transfer of materials from the solid phase to the gas phase via atom by atom or molecule by molecule to deposit the coating materials onto the fiber surface. Vacuum evaporation, ion implantation, and sputtering are three main techniques belonging to PVD. All of them can be used for preparing conductive fibers, and the last one is considered as an environment-friendly functional technology [113].

Vacuum evaporation is one of the most common methods for depositing functional films on various substrates. The vacuum prevents the evaporated particles from colliding with the background gas or other unwanted particles, allowing the vapor particles to be deposited directly onto the substrate. The vacuum evaporation process includes two steps: evaporation of the functional materials and condensation of them on the substrate. In vacuum evaporation, the coating material is melted, gasified, and evaporated by electric heating or electron beam heating. Then, the steam of the coating material reaches the substrate surface and gradually cools, eventually forming a film layer with good quality [114]. Wei Q et al. [115] fabricated a conductive fiber with good mechanical durability by first pretreating the gelled nylon, and then creating the coating with gold and silver in a classical 250 Pfizer vacuum system. Although vacuum evaporation is a simple way for manufacturing conductive fibers, it has a disadvantage of low bond strength between the coating and the substrate [116].

Ion implantation is a surface modificative process in which the ions from one material are implanted into another solid material to change the physicochemical properties of the material surface. For example, Elena et al. [117] prepared capillary bundle conductive fibers by modifying the inner surface of hollow polymer fiber bundles by plasma ion implantation (PBPII). During the process, an intact hollow fiber is put into the hollow cylindrical electrode collected with a negative high voltage pulse generator. In addition, nitrogen enters the hollow fiber by connecting the electrode inlet to a gas source. After applying a negative high voltage bias pulse to the electrode, the plasma is excited to achieve surface modification of the inner and outer surfaces of the hollow fiber.

**Figure 6:**
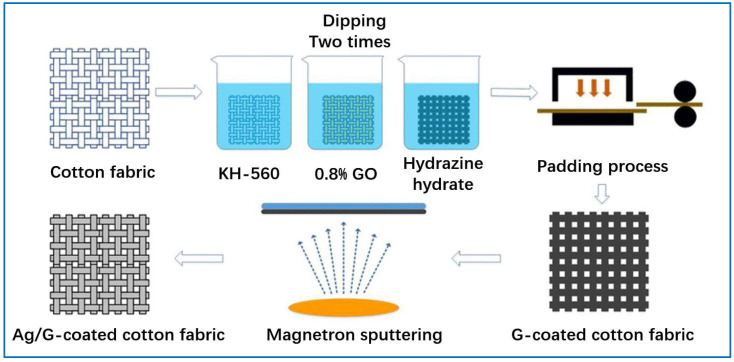
Preparation process of Ag/G-coated cotton fabric via PVD approach [70].

Sputtering is one of the most promising technologies in PVD, which first appeared in the late 1970s. It has been widely used in numerous areas for the modification of various materials. Sputtering is a process in which high-energy ions bombard the target, causing the atoms or molecules on the target to fall off. These ejected atoms or molecules agglomerate and form thin films on the substrate owing to their kinetic energy and orientation [118]. He et al. [70] used a magnetron sputtering system (model: MSP-300C) to prepare silver/graphene-coated cotton fabric. The vacuum chamber is evacuated and the graphene-coated cotton fabric prepared by the dip-coating method is fixed to the holder at a distance of 150 mm from the emission source, and, during the emission process, the holder rotates with the graphene-coated cotton fabric around its axis at a speed of 100 r/min so that the silver particles are uniformly deposited on its surface. The target product, silver/graphene-coated cotton fabric, is obtained with good flexibility and high electrical conductivity after electro-mechanical testing. Figure 6 shows the whole preparation process of Ag/G-coated fabric.

### 3.9. Wet Spinning

**Figure 7:**
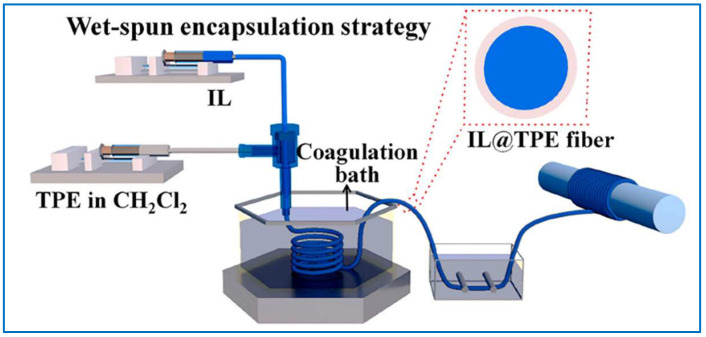
Schematic of preparation process of the IL@TPE fibers via wet spinning [128].

Wet spinning is a traditional fabricate technology, which has advantages such as simple process, low cost, and easy industrialization. In the process of wet spinning, the solution is injected into a coagulation bath through a microporous spinneret, and the water in the solution is removed through a coagulation bath to solidify the solution into fibers. Wet spinning can continuously prepare fibers with high electrical conductivity and flexibility, which can be used as wearable sensors for detecting strain [119,120,121,122], temperature [123], gas [124,125], optics [126], and pH [127]. Figure 7 shows a wet spinning process to prepare IL@TPE fiber [128]. Gao et al. [129] synthesized PEDOT: PSS and PVA conductive composite fibers by wet spinning. Furthermore, the conductivity of composite fibers can be improved after treatment with ethylene glycol (EG) solvent. The composite fibers exhibit good tensile recovery and electrical conductivity over a 20% strain range, which can be used as a wearable sensor for monitoring human motion and real-time health status. Chen et al. [130] prepared a core-shell structured conductive hydrogel/thermochromic elastomer fiber by wet spinning. The conductive hydrogel shows high conductivity and good tensile properties, and the thermochromic elastomer’s color inverts with the ambient temperature. The composite fibers are strain- and thermal-sensitive, which can be employed for monitoring human motion and detecting room temperature. Ugale et al. [131] fabricated highly ordered GO fibers by wet spinning, and then used hydrothermal assisted chemical to synthesize ZnO-modified ZrGO fibers. The sensors made of ZrGO fibers have low detection limits of 1.5 and 8 ppm for NO_2_ and H_2_S gases, respectively. So, this type of sensor can be used to detect the two toxic gases at room temperature.

### 3.10. Electrospinning

**Figure 8:**
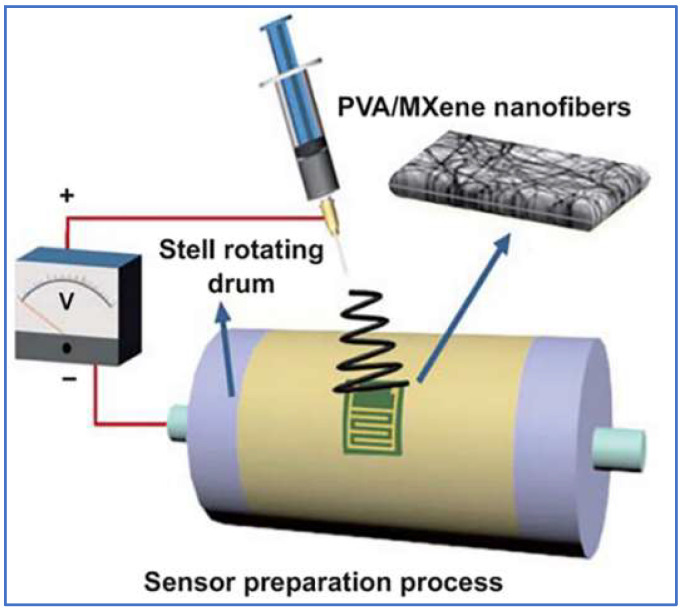
Schematic of preparation process of the PVA/MXene nanofibers via electrospinning [133].

Electrospinning is a simple and effective method that can fabricate micro-nanoscale fibers. The electrospinning device generally contains a high-voltage power supply, a syringe pump, a metallic needle, and a collector [132]. During the electrospinning process, the polymer solution will be pumped to the tip of a metallic needle, and the Taylor cone is formed under the effect of the electric field. When the electrostatic field force is stronger than the surface tension of the fluid, a charged jet ejects from the tip of the Taylor cone. Subsequently, the polymer jets are stretched and the polymer solution solvent will evaporate, and nanofibers are finally obtained on the collector. The electrospinning method provides more advantages than the other commonly adopted approaches to produce conductive nanofibers, such as solution-gel and CVD. An example of fabricating PVA/MXene conductive fibers via electrospinning is illustrated in Figure 8 [133].

Electrospun conductive nanofibers have a large specific surface area, high porosity, and excellent electrochemical properties. They can provide penetration channels and physical/chemical adsorption sites for electrons, which is helpful to improve the electrical properties of the materials. There are three main methods to fabricate electrospun functional nanofibers: (1) direct electrospinning with conductive polymers to obtain functional nanofibers; (2) direct electrospinning through polymer solution doped with conductive particles, such as Ag nanoparticles, graphene, and CNTs; or (3) fabricating nonconductive polymers into nanofibers by electrospinning, and then coating them with conductive materials by post-treatment [134]. Due to the flexibility of electrospun conductive fiber, it has application prospects in wearable sensors. Some recent examples of sensing applications based on electrospun conductive nanofibers are concluded in Table 2, indicating that the electrospun-fiber-based sensors have many advantages, such as high sensitivity and selectivity.

Because the traditional polymers are inherently insulators, electrospun polymer fibers are nonconductive [84]. In order to fabricate the conductive fibers, researchers usually use conductive polymers or polymer solutions doped with conductive materials as raw sources for electrospinning. Conductive polymers are obtained by chemical or electrochemical doping of polymers with conjugated π-bonds, which exhibit properties such as solution processability, high elasticity, high toughness, and low-temperature synthetic routes [138]. However, most conductive polymers have low molecular weight and low solubility in most solvents; only a few soluble conductive polymers can be directly electrospun into fibers. For example, Yu et al. [149] obtained a high-molecule-weight HCl/H_2_SO_4_-doped PANI solution by the magnetic stirring of the powder in hot sulfuric acid. The solution was electrospun into sub-microfibers collected by a coagulation bath. After dipping the PANI fibers in the bath for 24 h, the obtained fibers were pulled out and dried at room temperature. It was found that the PANI fibers with a diameter of 370 nm exhibit a high conductivity of 52.9 S/cm. In view of the fact that direct electrospinning with conductive polymers is complex, most researchers chose to electrospin polymer solutions doped with conductive materials to prepare multifunctional conductive fibers. Phuoc et al. [44] fabricated SnO_2_ porous nanofibers with SnCl_2_/PVP solution by one-step electrospinning. The prepared SnO_2_ nanofiber, with an average diameter of 150 nm, and the corresponding sensors indicate a good selectivity of H_2_S with short-term stability and a low detection limit. Qi and colleagues [63] obtained a stretchable and wearable sensor by electrospinning with CNTs/PU solution, which can be used to detect a variety of mechanical stimuli, such as pressure, stretching, and bending.

Another preparation method of conductive fiber is combining electrospinning with other techniques: first, electrospun nonconductive polymers, and then adding conductive materials to the nanofibers by spraying, chemical plating, in situ polymerization, and so on. This method is convenient to manufacture nanofibers with special structures and functionalities. Li et al. [150] electrospun with PAN/SiO_2_ solution into nanofibers, and then deposited AgNPs on the surface of nanofibers by chemical plating. The fibers with core-shell structure not only have a high electrical conductivity of 177.88 S/cm, but also exhibit good electrical conductivity stability in acid and alkaline solutions. Jia et al. [151] prepared a multifunctional RGO/TPU/ polydopamine (PDA) strain sensor with a three-dimensional network structure by combining electrospinning, ultrasound, and dopamine in situ polymerization methods. The sensor has excellent tensile properties, high sensitivity, fast response time, and good durability, which can be applied for real-time monitoring of human motions. In addition, the sensor shows good breathability expected of high-performance gas and humidity sensors.

The study of fiber’s microstructure is an important topic related to electrospun fibrous sensors. Due to the fiber microstructure being closely related with sensor function and performance, it is necessary to improve the electrospinning technology to obtain conductive fibers with smaller fiber diameter, larger specific surface area, and special structures (hollow and core-shell structures).

### 3.11. Other Ways

**Figure 9:**
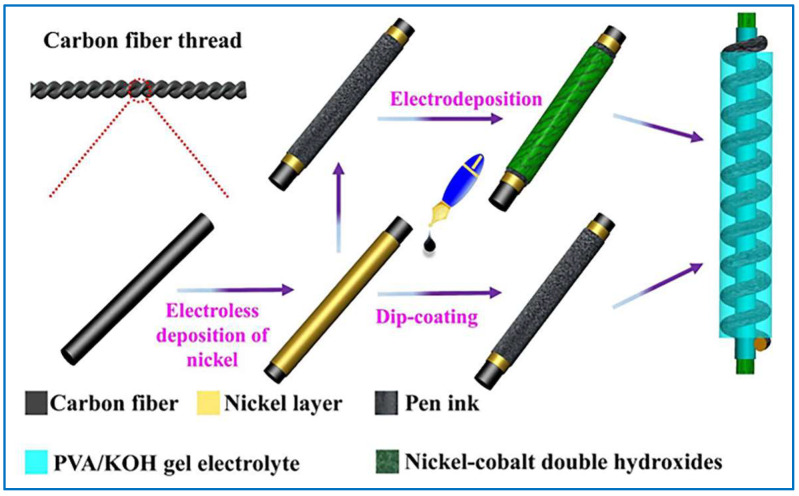
Schematic of preparation process of a flexible fiber-shaped supercapacitor by a combination of electroless deposition and dip-coating techniques [115].

In addition to the above methods, there are also diverse ways to prepare conductive fibers. For example, Souri et al. used the ultrasonic method to prepare conductive fiber yarn. First, a uniform conductive dispersion is prepared, and then the yarn is put into an ultrasonic bath for the coating process [62]. Seyedin et al. [152] have also obtained conductive MXene-polymer composite fibers by treating MXene with a solvent exchange (SE) method. The SE method is direct, ultrasonic-free, and highly versatile because multiple solvent transfers can be performed sequentially in multiple solvents to generate MXene. Park S et al. [153] have injected liquid gallium into hollow elastomer fibers composed of triblock copolymer poly[styrene-b-(ethylene-butylene)-b-styrene] (SEBS), using liquid metal to change temperature for solidification. In the end, elastomeric conductive fibers with shape memory properties are produced by liquid state conversion. It is known that different ways have different advantages, so that sometimes multiple methods are combined to prepare multifunctional conductive composite fibers. For example, Liu et al. first used the in situ polymerization method to polymerize the dopamine monomer on the surface of the glass fiber, and then applied the electroless plating approach to coat a layer of silver to obtain the polydopamine/silver conductive composite fibers. The silver layer on the surface is dense, uniform, continuous, and in the state of metal crystalline, which shows a very good electrical conductivity [154]. In another study by Lu et al., a flexible fiber-shaped supercapacitor based on nickel–cobalt double hydroxide and pen ink electrodes on metallized carbon fiber are manufactured by electroless deposition and dip-coating techniques, as shown in Figure 9 [115]. With the advancement of science and technology, superior and better conductive fibers will appear in the future, which can be prepared into various high-performance wearable sensors. 

## 4. Conductive-Fiber-Based Sensors 

**Figure 10:**
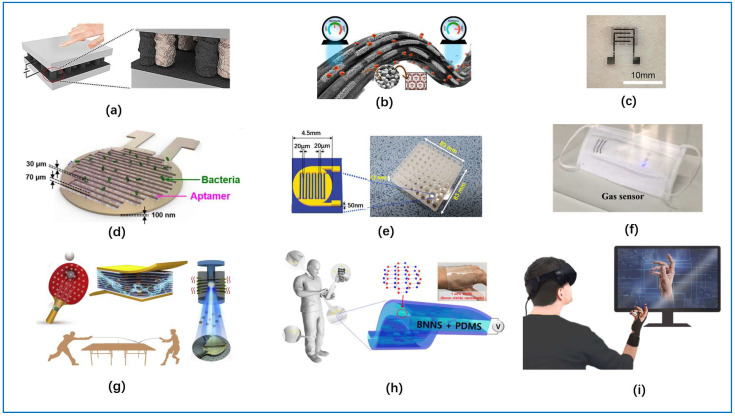
A wide variety of sensors are prepared from conductive fibers. (**a**) Flexible piezoresistive sensor based on interlocking graphene microarray, (**b**) a humidity sensor prepared using a metal-organic framework (MOF) and a fiber fabric, (**c**) photography of temperature sensor attached on skin, (**d**) schematic of aptamer-functionalized capacitance sensor, (**e**) schematic of aptamer-functionalized capacitance sensor, (**f**) a multi-functional face mask with integrated fiber gas sensors, (**g**) piezoelectric sensor made into sports training system, (**h**) transparent and flexible piezoelectric human motion sensor, (**i**) a glove for user–VR interaction (piezoelectric sensor) [158,159,160,161,162,163,164,165,166].

The applications of flexible and stretchable electronics in engineering technologies have made possible the fabrication of slender, lightweight, stretchable, and foldable sensors [3]. Among all electronic technologies, the most mature and successful are the fiber-based sensors, which have been practically used in wearable sensors [15]. The role of a sensor is to sense and convert stimulus into a signal measured by electronic components when a specific target is stimulated [95,155]. Wearable sensors based on conductive fibers are very good at conveying a variety of information from the human body, where the most popular includes pressure sensors, strain sensors, moisture-sensitive sensors, and gas-sensitive sensors [153,156,157]. Among them, pressure sensors and strain sensors are the main ones. As shown in Figure 10, a wide variety of wearable sensors are manufactured with conductive fibers.

### 4.1. Pressure Sensors

**Figure 11:**
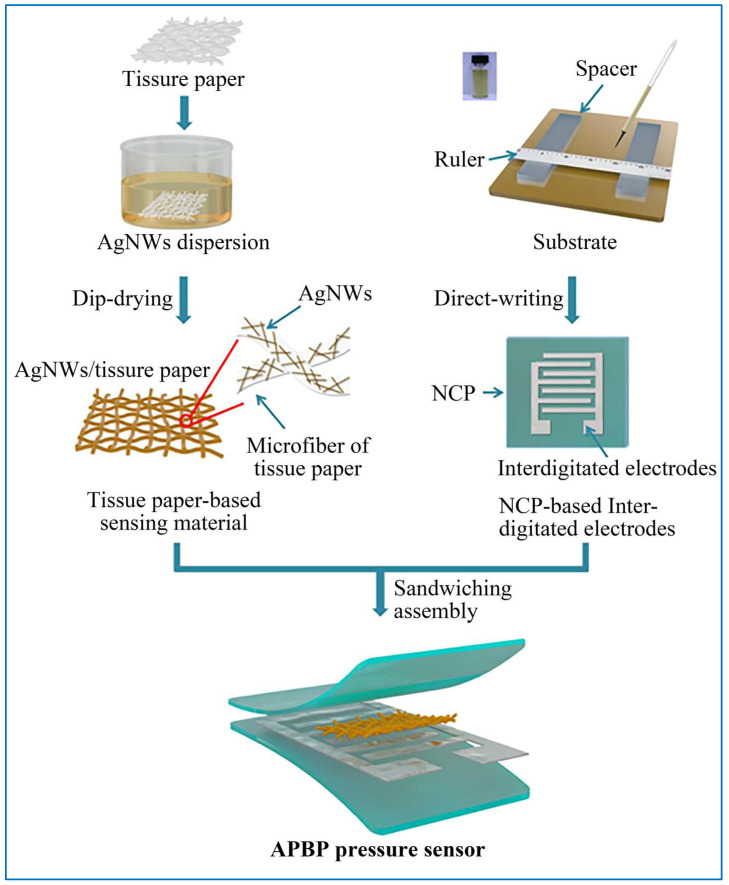
Schematic of preparation process of an all paper-based flexible and wearable piezoresistive pressure sensor [169].

Pressure sensors, also known as tactile/tension sensors, have attracted much attention in recent years owing to their unique merits, such as mechanical compliance, fast response, high sensitivity, durability, biocompatibility, as well as being lightweight. It can be closely attached to human skin to monitor physiological health conditions, such as heart rates or breathing patterns, in real time [110]. Usually, pressure sensors can convert mechanical signals of external pressure into electrical signals by means of converting mechanical stimulus into electric signals, including resistance, capacity, and voltage, resulting in three types of pressure sensors: resistive sensors, capacitive sensors, and piezoelectric sensors [167,168]. A common preparation process of a pressure sensor is shown in Figure 11, where an all paper-based flexible and wearable piezoresistive pressure sensor is prepared by Gao et al. [169].

#### 4.1.1. Resistive Pressure Sensors

A resistive sensor, also named piezoresistive sensor, converts a resistance change of a sensitive material caused by an external stimulus into an electrical signal output [11]. When the sensor is under pressure, the resistance becomes larger according to the law of resistance:

(1) $$R=\frac{\rho L}{S}$$

where *ρ*, *L*, and *S* are the electrical conductivity, length, and cross-sectional area of the material, respectively. From Ohm’s law:

(2) V=IR

where *V*, *I*, and *R* are the voltage, the current, and the object’s resistance. As the current is constant, a higher resistance leads to a larger output voltage [120]. Commonly, this kind of sensor with simple internal structures and circuitry shows a low manufacturing cost, high sensitivity, good linearity, and long durability. In the process of applications, it can quickly and accurately measure and record the pressure not only at rest, but also in real time [169]. Yue et al. prepared a highly sensitive piezoresistive sensor by using MXene-sponge and PVA nanowires. After 10,000 cycles of loading, the sensor can still maintain good stability, and the sensitivity value remains more than 90% of the original value. The minimum detectable pressure is 9 Pa and the short fast response time is 138 ms, which allows real-time monitoring of human physiological signals (e.g., breathing, joint motion, and pulse) [170]. Similarly, Yang et al. [171] also utilized MXene to prepare piezoresistive sensors with micro-spinous microstructures, which possess high sensitivity (151.4 kPa^−1^), short response time (less than 130 ms), large pressure detection limit (up to 4.4 Pa), and good cycling stability (more than 10,000 cycles). Inspired by ultra-light and structurally robust spider webs, Liu et al. [172] used conductive polyimide nanofibers to fabricate resistive pressure sensors with excellent properties of low density (9.98 mg cm^−1^), normal operating temperature range (−50–250 °C), excellent compressibility and recoverability (up to 90% of strain), superior fatigue resistance over 1000 cycles, etc. In particular, the resistive pressure sensor possesses a good oil–water separation characteristic due to its hydrophobic and robust graded porous structure. 

From the above discussion, it can be seen that these resistive pressure sensors prepared from conductive fibers are characterized by high sensitivity, fast response, good durability, and excellent wearability, which can be well used to monitor human health, movement conditions, and so on. Table 3 shows the conductive-fiber-based resistive pressure sensors with different properties. From the table, it is obvious that the working range, sensitivity, durability, and response time of these sensors are relatively wide, high, good, and short, respectively.

#### 4.1.2. Capacitive Pressure Sensors

Capacitive sensors can respond to external force change through capacitance variation, and typically consist of two parallel flexible conductive planes separated by a dielectric layer. Capacitance (*C*) is given by the equation:

(3) $$C=\frac{\varepsilon_{0}A}{d}$$

where *d*, *A*, and $\varepsilon_{0}$ are the distance between the upper and lower electrodes, the effective area, and the dielectric constant of the dielectric layer, respectively. With a constant effective area of the conducting plate, the capacitance varies with the distance between them [183,184]. In general, the process of preparing a capacitive sensor with conductive fibers is usually accomplished by placing the dielectric into a flexible material to form a cylindrical capacitance. When the sensor is subjected to pressure, the capacitance increases with the decreased distance between the conductive plates and increased effective area, thus converting the mechanical signal into an electrical signal. 

PDMS polymer is often used as a good flexible material to prepare flexible sensors. A new flexible capacitive pressure sensor with high sensitivity of 0.11%/MPa for capacitance change and a correlation coefficient of 0.9975 was prepared by Ali et al., which employs PDMS as the outer flexible layer and is then filled with EGaIn liquid metal, followed by corona discharge treatment [185]. In another study, Chhetry et al. have fabricated a flexible, highly sensitive capacitive pressure sensor by coating microporous PDMS elastomeric media on conductive Twaron fibers, which shows relatively high sensitivity of 0.278 kPa^−1^, low detection limit of 38.82 Pa, and good durability for more than 10,000 cycles [186]. Geng and colleagues made flexible stretchable cylindrical capacitive pressure sensors by combining thermoplastic elastomer composites with carbon black conductive additives, which display highly desirable characteristics, including fast response, low hysteresis, high sensitivity, and signal stability during static and dynamic stresses [187]. Zheng et al. [188] have developed a flexible capacitive pressure sensor based on reduced graphene oxide cotton fibers through a simple, low-cost preparation process. Not only does it have ultra-high sensitivity (up to 15.84 kPa^−1^) and wide sensing range (0–500 kPa), but it also has excellent durability (more than 400 cycles), low hysteresis (≤11.6%), low detection limit (<0.1 kPa), and wide frequency availability (sensitivity from 19.71 to 11.24 kPa^−1^). The sensor can detect various external stimuli (vertical stress, bending, and airflow) and has been successfully applied to facial expression recognition, respiration detection, joint motion, and walking detection, indicating a great potential application in artificial electronic skin and wearable medical devices. Table 4 shows the conductive-fiber-based capacitive pressure sensors with different properties.

#### 4.1.3. Piezoelectric Pressure Sensors

The piezoelectric pressure sensor is a self-powered device with high sensitivity and good durability, which depends on piezoelectric materials. A normal piezoelectric material can generate electrical signals in response to the externally applied load, which is called the piezoelectric effect. It arises from variation in the arrangement of ions in a piezoelectric material once subjected to stress, and the neutral of the charges in the crystal begins to break, resulting in an electric field at the material boundary [195]. Piezoelectric pressure sensors take advantage of this property to convert pressure signals into electrical signals like voltage. The use of conductive fibers to prepare piezoelectric pressure sensors is not very common. PVDF, a thermoplastic and semi-crystalline polymer, is distinguished in the field of piezoelectric pressure sensors owing to its good piezoelectric properties under certain conditions. Hofmann et al. [196] prepared a braided piezoelectric pressure sensor using three types of fibers: PVDF multifilament yarn, high tenacity polyester PES filament (PHP Diolen^®^174S), and PA yarn, of which PES filament is used as a reinforcing and insulating material. The dynamic test results show that the sensor has a high sensitivity. Negar et al. [197] also applied PVDF nanocomposite fibers to fabricate flexible piezoelectric pressure sensors with enhanced performance by doping PZT particles (PbZr_1-x_Ti_x_O_3_). Fatemeh et al. [53] used a similar way to prepare PVDF hybrid fibers with piezoelectric function by melt spinning BT nanoparticles mixed with PVDF (mass ratio of 1:10). The resulting piezoelectric pressure sensor can also be a good energy harvester. In addition, Sathiyanathan et al. prepared a piezoelectric pressure sensor with PVDF and poly (octafluoropentyl acrylate) (POFPA) blended nanofibers filled with sulfonated poly (phenyl sulfide) (sPPS) as a filler, which shows an excellent sensing performance [198]. Table 5 shows the conductive-fiber-based piezoelectric pressure sensors with different properties.

### 4.2. Strain Sensors

**Figure 12:**
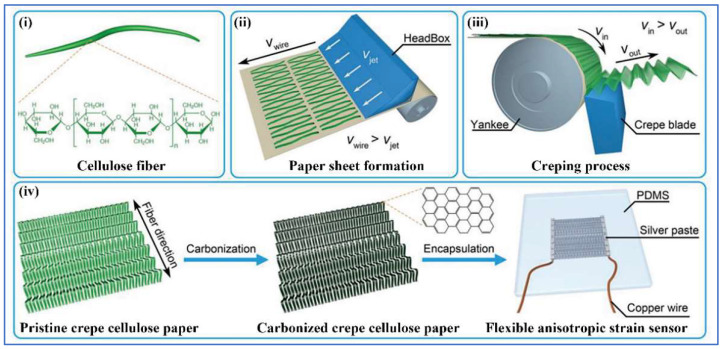
Schematic of preparation process of a flexible and anisotropic strain sensor. (**i**–**iii**) Schematic illustration showing the fabrication process of crepe cellulose paper with aligned cellulose fibers. (**iv**) Schematic illustration showing the fabrication process of the flexible CCP strain sensor [208].

Conductive fibers have good stretchability and flexibility, so they are well-suited to be prepared as strain sensors, especially resistive strain sensors. The principle of the strain sensor is to convert the mechanical signal into an electrical signal by the change in resistance caused by stretching. Metal-based conductive fibers, including liquid metals and silver, are often used as strain sensors due to their high conductivity, ductility, and stability. Zhang et al. [96] used EGaIn as well as silicone rubber filaments to fabricate conductive fibers with high tensility and electrical conductivity. The final strain sensor prepared using the conductive fibers has a large deformation and good stability. Chen et al. [16] also used liquid metals to prepare a resistive strain sensor with high ductility, conductivity (over 10^3^ S cm^−1^), and resistance to high temperatures (remaining stable at temperatures close to 250 °C), except that they replaced rubber filaments with PU fibers. Using silver nanoparticles and fibers with stretchable structures, Lee et al. [34] have manufactured a resistive strain sensor with excellent stretchability, high sensitivity, wide sensing range, and good durability. Although carbon materials are generally not highly conductive, some of them, such as CB and CNT, have good conductivity. Li et al. [206] have prepared a paper-based (PB) resistive strain sensor with excellent durability, superior waterproofing, and good sustainability, which is capable of detecting ultra-low strains due to the presence of ultra-sensitive microcrack structures in the conductive CB/CNT layer. Moreover, the sensor has a strain coefficient of 7.5 in the tensile strain range of 0–0.7%, which is almost three times higher than that of conventional metal-based sensors. Similarly, Fan et al. [207] prepared a resistive strain sensor with excellent properties using carbon-based conductive fibers. Xu et al. also prepared a flexible and anisotropic strain sensor with carbonized crepe paper and aligned cellulose fibers, and the fabrication procedure is shown in Figure 12 [208]. In another study, Wang et al. [209] used conductive poly(styrene-butadiene) composite fibers to fabricate a highly sensitive, stretchable resistive strain sensor with the superior performance of wide strain range (>110%), high sensitivity, and durability. There are also many conductive fibers prepared as strain sensors with a large strain range, high sensitivity, and good durability, and their characteristics are listed in Table 6. 

### 4.3. Other Sensors

Conductive fibers are mainly prepared as mechanical sensors (pressure sensors and strain sensor), but some can also be made as gas sensors, humidity sensors, etc. By absorbing moisture in the air, the capacitive or resistive values of conductive fibers with moisture-sensitive materials (PVA, polyimide, CNT, etc.) would change, resulting in different humidity response signals. This is the mechanism of humidity sensors, which can be classified into two types: resistive humidity sensors sensor and capacitive humidity sensors sensor. Ma et al. [218] winded a moisture-sensitive fiber as a capacitive dielectric layer around a copper wire to prepare a fiber-based humidity sensor with stable humidity response performance. Shahzad et al. [219] have used stainless steel fibers with good moisture absorption properties to prepare moisture or trauma exudate detection sensors for monitoring bedridden, disabled, or ulcerated patients, where the applied fibers’ conductivity increases with the relative humidity. After studying various materials, the researchers discovered that PANI can be a good candidate for making a gas sensor owing to its high sensitivity to gases, low response time, excellent stability, and low cost. For example, Wang et al. [220] reported that they used PANI nanofibers to prepare a gas sensor with high sensitivity and fast response time, and its sensing performance can be tunable by the polymerization temperature and the acid concentration during the preparation process.

## 5. Summary and Outlook

This paper briefly reviews the research progress of conductive fibers and their applications in sensors in recent years. First, the properties of different types of materials made for conductive fibers are concluded. Then, various preparation strategies of some conductive fibers are described in detail. Finally, several kinds of sensors prepared from conductive fibers are analyzed. The encouraging achievements highlighted in this paper show that conductive fibers are soft, deformable, breathable, durable, and washable, and that the sensors prepared from them have high sensitivity, wide measurement range, good stability, and comfortable wearability [221,222].

In the future, conductive fibers are promising to become the material choices for wearable sensors. Because the conductive fiber not only inherits the softness, breathability, stretchability, weaveability, and other characteristics of the fiber, but also possesses a long-lasting high conductivity, stability, good electromechanical responsiveness, and other properties, all of the properties of the mentioned conductive fibers are very suitable as the raw materials for wearable sensors. Despite the considerable advantages of conductive fibers, further improvements are needed. Future progress may be made in the following areas: (1) deep research on conductive fiber materials and preparation strategies to develop more lightweight, eco-friendly, multi-functional integrated conductive fibers; (2) intensive employments of conductive fibers to develop a more sensitive, extensive, and comfortable multifunctional wearable artificial intelligence sensor, which is capable of monitoring various health parameters of the human body in real time, transmitted to themselves, communities, and hospitals through the internet to prevent diseases.

## Figures and Tables

**Table 1:** Performance parameters of some conductive fibers.

Conductive Fiber Category	Principle Material	Indicators			Application	Ref.
		Electrical Conductivity (S cm^−1^)	Repeatability (Cycles)	Response Time (ms)		
Metal-based conductive fibers	Ag nanoparticles (AgNPs)	6.99	>10^4^	<340	Capacitive pressure sensor	[36]
	Gallium-indium alloy (EGaIn)	10^3^	8 × 10^3^	220	Strain sensor	[37]
	MXene			50	Piezoresistive pressure sensor	[38]
	Silver nanowires (AgNWs)	3.1	2.5 × 10^3^	49	Resistive strain sensor	[39]
	Barium titanate (BT) nanoparticle		10^3^	2000	Resistive strain sensor	[40]
	Nickel (Ni)	20	2 × 10^3^		Resistive strain sensor	[41]
Carbon-based conductive fibers	Multi-walled carbon nanotubes (MWCNTs)		10^4^		Resistive strain sensor	[42]
	Graphene nanoplates (GPs)	280.8	2 × 10^3^		Resistive strain sensor, temperature sensor, humidity sensor	[43]
	Graphene (G)		10^3^	33	Resistive strain sensor	[44]
	Carbon black (CB)	5.8	10^3^		Resistive strain sensor	[45]
Polymer-based conductive fibers	Polyaniline (PANI)	3.72 × 10^−4^			Gas sensor	[46]
	Poly(3,4-ethylenedioxythiophene) (PEDOT: PSS)	120			Piezoresistive pressure sensor	[47]
	Poly (ethylene glycol) (PEG)	0.765	10^3^		Resistive strain sensor	[16]
	Polypyrrole (PPy)		10^3^	20	Piezoresistive pressure sensor	[48]

**Table 2:** Examples of sensing applications of electrospun conductive nanofibers.

Category	Materials	Properties	Sensing Type	Reference
Metal-based	Metal NPs	High sensitivity, selective and flexible, low limit of detection	Gas	[135]
	Metal oxide	High sensitivity, good stability and low detection limit	Gas	[136]
	MXene	Fast response and recovery, good flexibility and reliability	Gas	[137]
		Wide sensing range, high sensitivity	Strain	[138]
		High sensitivity, fast response time, good stability	Pressure	[139]
		Flexible, self-powered	Humidity	[140]
Carbon-based	Graphene	Flexible, highly sensitive	Pressure	[141]
	RGO	High sensitivity, short response time and good cycle stability	Gas	[142]
	CNTs	Stretchable and multimodal	Strain/pressure	[143]
	CB	High sensitivity, good stability and durability	Strain	[144]
Polymer-based	PANI	Highly sensitive, good sensing linearity, fast Response, small and good repeatability	Humidity	[145]
		Highly flexible, sensitive and stability	PH	[146]
	PPy	High sensitivity, fast response and recovery	Gas	[147]
	PEDOT: PSS	Highly flexible, stretchable	Strain	[148]

**Table 3:** Performance data sheets for some resistive pressure sensors.

Main Component Materials of Conductive Fibers	Working Range	Sensitivity (KPa^−1^)	Durability (Cycles)	Response Time (ms)	Ref.
CNTs		136.8, *p* < 200 Pa			[173]
G	1.0 Pa–32 kPa	10.41, *p* < 2.5 kPa	>10^4^	<19	[174]
PANI foam	>4 pa	0.055, 0 < *p* < 5.0 kPa	>5 × 10^2^	60	[175]
AgNWs	30–30,247 pa	1.5, 0.03 < *p* < 30.2 kPa		90	[176]
5CNTs		1150.9, *p* < 50 Pa	> 2 × 10^3^	43	[177]
Ink-modified plant fiber sponges (m-PFS)	10–750 Pa	133.3, 10 < *p* < 750 Pa			[178]
Graphene, P(VDF-TrFE)	<45 mmHg	0.76, *p* < 45 mmHg		<100	[179]
MWCNTs		66.7%−1, 160 mN < *p* < 300 mN	10^3^	<200	[180]
Wrinkled graphene foams			>10^5^	150	[181]
MWCNTs, PDMS		2.65		80	[182]

**Table 4:** Performance data sheets for some capacitive pressure sensors.

Main Component Materials of Conductive Fibers	Working Range (kPa)	Sensitivity (kPa^−1^)	Durability (Cycles)	Response Time (ms)	Ref.
AgNPs, PDMS	0–10	0.278 (*p* < 2 kPa)	10^4^	<340	[56]
CNTs, Silver paste	0–1000		2 × 10^4^	7	[189]
Graphite, carbon fiber	0–40	0.045	10^3^	135	[190]
MXene (Ti_3_C_2_T_x_)	<400	0.51	10^4^		[191]
PVA, potassium hydroxide (KOH)		20.83	1.6 × 10^3^	50	[192]
AgNWs, PDMS	<200	0.161 (*p* < 10 kPa)	6 × 10^3^		[193]
CNTs, PDMS		0.17	5 × 10^3^	25	[194]

**Table 5:** Performance data sheets for some piezoelectric pressure sensors.

Main Component Materials of Conductive Fibers	Applied Force(N)	Sensitivity (V/N)	Output Voltage (V)	Durability (Cycles)	Ref.
BT, PVDF, polydopamine (PDA)	0.22–19.33	0.38		6.834 × 10^3^	[199]
BTO, PVDF, PDA		3.95		7.4 × 10^3^	[200]
Platinum (Pt) NPs, PVDF	<20	0.6	30	9 × 10^4^	[201]
Poly (vinydene fluoride-trifluoroethylene) (P(VDF-TrFE)), Cu		0.068	0.8	1.5 × 10^4^	[202]
PVDF, BaTiO3			0.1	1.75 × 10^3^	[203]
PVDF, MOF	<5	0.118	0.568		[204]
Lead zirconatetitanate (PZT)	>0.05	8.117			[205]

**Table 6:** Performance data sheets for selected resistive strain sensors.

Main Component Materials of Conductive Fibers	Working Range	Gauge Factor (GF)	Response Time (ms)	Durability (Cycles)	Ref.
G	<15%	GF = 1.7			[210]
Thermoplastic polyurethane (TPU) fibrous	<300%	100%, GF = 428.5 100–220%, GF = 9268.8 220–300%, GF = 83,982.8	70	>10^4^	[211]
Carbonized crepe paper (CCP)	<5%		<115	>10^4^	[212]
Cellulose nanocrystal (CNC), G	<98%	GF = 2.36 × 104	33	>10^3^	[65]
Overlapped carbon nanotubes	≥145%	125–145%, GF = 42,300			[213]
CNTs	>700%	250–700%, GF = 3.39	300	>300	[214]
Cotton fabrics, graphene nanosheets, PDMS			90	10^4^	[215]
AgNWs	60%	GF = 1.5			[216]
Ti_3_C_2_T_x_MXene, CNTs	130%	GF = 772.6		>5 × 10^3^	[217]

## Data Availability

Not applicable.
